# Deterministic and stochastic approaches in the clinical application of mesenchymal stromal cells (MSCs)

**DOI:** 10.3389/fcell.2014.00050

**Published:** 2014-09-12

**Authors:** Simone Pacini

**Affiliations:** Department of Clinical and Experimental Medicine, University of PisaPisa, Italy

**Keywords:** mesenchymal stromal cells, cell-based therapy, clinical applications, cell culture techniques, risk assessment, mesenchymal stem cell transplantation, bioreactors, cell isolation

## Abstract

Mesenchymal stromal cells (MSCs) have enormous intrinsic clinical value due to their multi-lineage differentiation capacity, support of hemopoiesis, immunoregulation and growth factors/cytokines secretion. MSCs have thus been the object of extensive research for decades. After completion of many pre-clinical and clinical trials, MSC-based therapy is now facing a challenging phase. Several clinical trials have reported moderate, non-durable benefits, which caused initial enthusiasm to wane, and indicated an urgent need to optimize the efficacy of therapeutic, platform-enhancing MSC-based treatment. Recent investigations suggest the presence of multiple *in vivo* MSC ancestors in a wide range of tissues, which contribute to the heterogeneity of the starting material for the expansion of MSCs. This variability in the MSC culture-initiating cell population, together with the different types of enrichment/isolation and cultivation protocols applied, are hampering progress in the definition of MSC-based therapies. International regulatory statements require a precise risk/benefit analysis, ensuring the safety and efficacy of treatments. GMP validation allows for quality certification, but the prediction of a clinical outcome after MSC-based therapy is correlated not only to the possible morbidity derived by cell production process, but also to the biology of the MSCs themselves, which is highly sensible to unpredictable fluctuation of isolating and culture conditions. Risk exposure and efficacy of MSC-based therapies should be evaluated by pre-clinical studies, but the batch-to-batch variability of the final medicinal product could significantly limit the predictability of these studies. The future success of MSC-based therapies could lie not only in rational optimization of therapeutic strategies, but also in a stochastic approach during the assessment of benefit and risk factors.

## Introduction

In recent years, human mesenchymal stromal cells (MSCs) have been extensively researched in clinical trials for the treatment of various bone/articular, immune, neurological, cardiovascular, gastrointestinal, and blood pathologies (http://clinicaltrials.gov). The clinical appeal of MSCs is mainly due to their easy and inexpensive isolation from many different tissues, as well as their lack of significant immunogenicity (Le Blanc et al., 2003). The enormous biological value of MSCs is derived by their differentiation ability, their immunoregolatory functions and their production of multiple paracrine growth factors and cytokines (Bianco et al., 2001; Horwitz et al., 2006; Tyndall et al., 2007; Shabbir et al., 2009). The MSC clinical value is further increased by the possibility that all of these various and important functions, could act sinergically administrating a unique cell population. Thus, clinical applications that, for instance, deal with tissue regeneration basing on the differentiation potential of MSC (Horwitz et al., 1999; Muguruma et al., 2006), could also take advantage by their reported support of neo-angiogenesis, to re-vascularize the new formed tissue (Cao et al., 2005; Au et al., 2008), as well as immunoregolatory function could modulate inflammation on the injured site (Glennie et al., 2005; Ringden et al., 2006). The downside of using MSCs for clinical purposes is that they occur in very low frequency in the tissue of origin, which forces the use of *in vitro* expansion protocols in order to achieve a significant number of cells that are feasible for transplantation.

MSC-based therapy is currently facing a challenging phase following the completion of many pre-clinical and clinical trials. Several trials reported moderate, non-durable benefits, which caused initial enthusiasm to wane, and indicated an urgent need to optimize the efficacy of therapeutic, platform-enhancing MSC-based treatment (Allison, 2009; Malliaras et al., 2011; Tyndall, 2011). The future success of MSC-based therapy lies in rational optimization of therapeutic strategies, in conjunction with an adequate assessment of benefit and risk factors (Liras, 2010).

In this review, emerging concepts on MSC identity, properties and physiological role (Keating, 2012) are discussed in correlation to important ethical principles and regulatory issues, about clinical use of these cells. In particular, the evidences of multiple origins of MSC in the organisms and the characteristic heterogeneity of culture expanded MSCs are indicated preventing a correct risks/benefits evaluation. As a consequence of MSC multiple origins and heterogeneity, their production results a high sensible process influenced by a large number of variables that could be predetermined only in part. In fact, unpredictable fluctuations in the environmental parameters at the time of sampling and/or during cell manipulation could significantly affect the final cell product biology. The main critique of this review discuss the general approach to clinical grade MSC production that still consider “crude” cell suspension as staring materials (i.e., bone marrow mononuclear cells or stromal vascular fraction) and uncontrolled culture conditions.

## Major principles and guidelines regulating clinical use of MSCs

### Precautionary principles (PP)

In existing literature, as well as in international treaties and declarations, a variety of PP definitions can be found. It is generally provided the following widely accepted PP definition: *“The precautionary principle or precautionary approach states that if an action or policy has a suspected risk of causing harm to the public or to the environment, in the absence of scientific consensus that the action or policy is harmful, the burden of proof that it is not harmful falls on those taking the action.”* In the year 2000, the European Union (EU) issued a communication regarding the definition of PP: “*The precautionary principle applies where scientific evidence is insufficient, inconclusive or uncertain and preliminary scientific evaluation indicates that there are reasonable grounds for concern that the potentially dangerous effects on the environment, human, animal or plant health may be inconsistent with the high level of protection chosen by the EU.”* Under European Union Law, application of the precautionary principle has been made a statutory requirement. The EU definition, on the other hand, requires intervention to maintain the high level of protection chosen by the EU. Moreover, the EU definition includes two main concepts: the “uncertainty of scientific evidence” and the “potentially dangerous effects.” These concepts give rise to the general principle that should be applied in clinical trial design and peer-review, and which can be summarized as “*ensure the safest possible method*” (COM.EST, 2005).

### European union (EU) regulatory issues

In order to applying PP in the definition of new therapies, assuring quality, safety and efficacy the European Union (EU) proposed a plan of action for the development of new biotech medicines. This plan included the definition of Advanced Therapy Medicinal Products (ATMPs) as a new medicinal product category that have to fulfill the same scientific and regulatory standards defined for all the other medicinal product as drugs and transplants.

**Figure 1:**
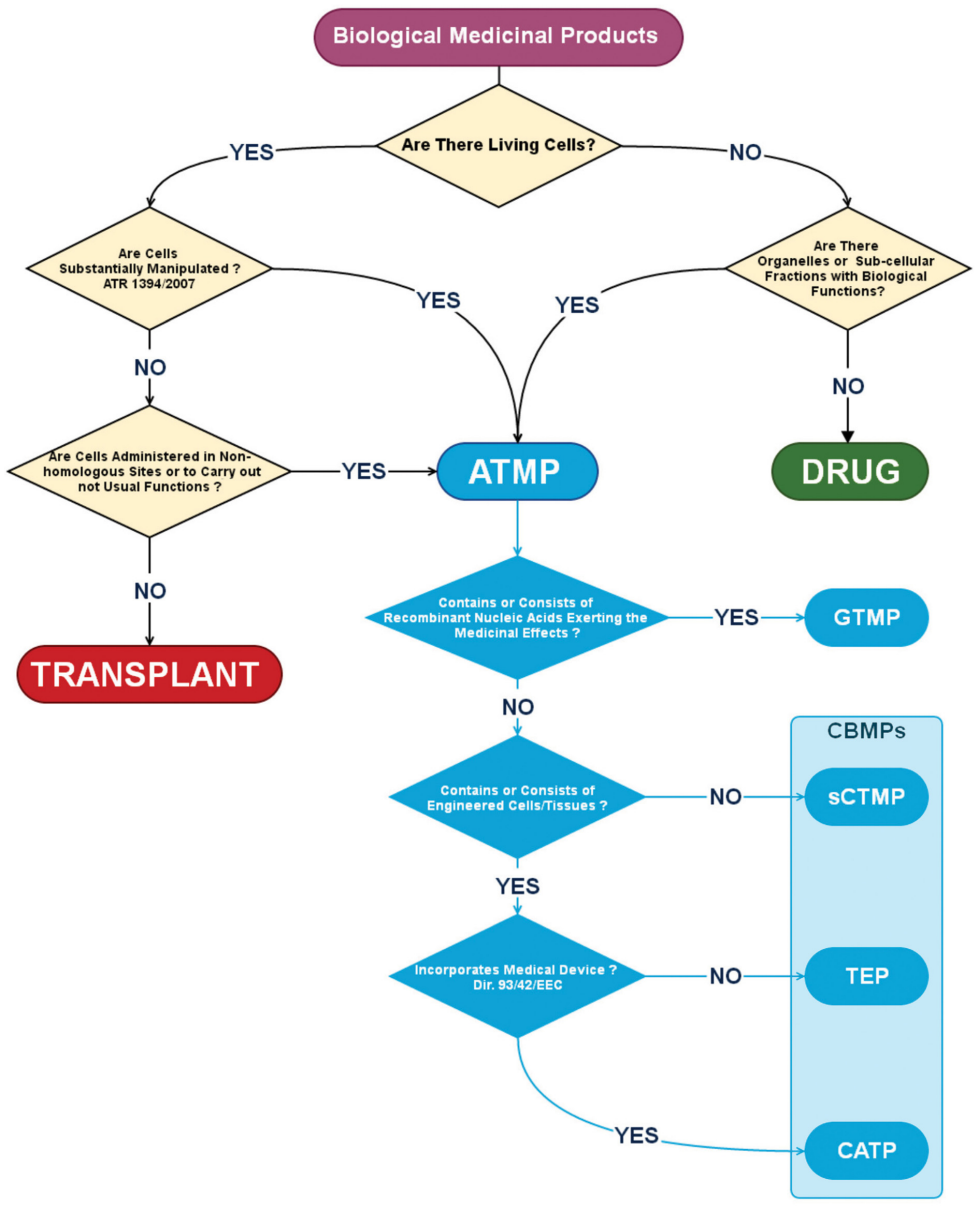
**Algorithm for the definition of a Cell-Based Medicinal Product (CBMP)**. According to the European Medicines Agency (EMA) a *Biological Medicinal Product* should be considered an *Advanced Therapy Medicinal Product* (ATMP) if contains, or consist of living cells undergoing substantial manipulation and/or administered in non-homologous sites. ATMP includes also medicinal products in which the therapeutic effect is carried out by organelles or sub-cellular fraction like in *Gene Therapy Medicinal Products* (GTMP) that contain or consist of recombinant nucleic acids. Other ATMPs have been defined as *Cell-Based Medicinal Products* (CBMPs, pale blue box) due to the presence of living cells alone as in *Somatic Cell Therapy Medicinal Product* (sCTMP), integrated in bioengineered composites as in *Tissue Engineered Products* (TEP) or in combination with other medical devices as in *Combined Advanced Therapy Products* (CATP).

A long succession of Directives and Community Regulations, reviewed in Gálvez et al. (2013), are effective from January the 1st of 2013, and governs the authorization, supervision, release and pharmacovigilance of AMTPs in EU. AMTPs have been defined as biological medicinal products containing or consisting of living cells as well as sub-cellular fractions with biological functions. Thus, AMTPs could not be included in the same category of drugs and differ from transplants because: (1) They contain *viable human cells* of allogeneic or autologous origin undergoing a manufacturing process including substantial manipulations (as defined in the Regulation (EC) n. 1394/2007, Annex 1); (2) They may be applied for non-homologous use. Noteworthy, definition of AMTPs is dependent on not only to the production process but also on their application, considering the concept of “non-homologous use” which means that cells are administered in sites where they are not usually present, or to carry out biological functions that they do not usually take part in (Figure 1). According to the European regulation, ATMPs include four different typologies of products: *gene therapy medicinal products* (GTMP); *somatic cell therapy medicinal products* (sCTMP); *tissue-engineered products* (TEP) and *combined advanced therapy products* (CATP) (Schneider et al., 2010). Briefly, GTMP differ from other AMTPs because they do not include living cells or tissue, and their medicinal effects are exerted by recombinant nucleic acids. On the contrary, the other AMTPs consist or contain living cells or tissue in form of cellular preparations (sCTMP), engineered products (TEP) or in combination with other medical devices (CATP, Figure 1). These latest products could be also gathered under the more generic definition of *Cell-based Medicinal Products* (CBMPs), where CBMPs are defined as: “*medicinal products presented as having properties for, or used in or administered to, human beings with a view to treating, preventing or diagnosing a disease in which the pharmacological, immunological or metabolic actions are carried out by cells or tissues*” (Figure 1, pale blue boxed).

As MSCs represent a substantially manipulated cell population, due to the culture expansion process, therapeutic products including these cells, should be classified as ATMPs. More specifically, MSC-based products should be included in the definition of CBMPs, as MSCs could be possibly applied as cellular suspension (in sCTMP), as cellular component in engineered biomaterials (in TEP) or in combination with other medical devices like artificial prosthesis, coronary stents, pacemaker leads, etc. (in CATP).

### Good manufacturing practices

In order to prevent any unnecessary and undesired risk exposure related to cell preparation, storage and transportation procedures, in 1997 the World Health Organization (W.H.O.) defined Good Manufacturing Practices (GMP) as “*that part of quality assurance which ensures that products are consistently produced and controlled to the quality standards appropriate to their intended use and as required by the marketing authorization*” (W.H.O, 1997). GMP covers all aspects of the manufacturing process. This includes the defined manufacturing process, validated critical manufacturing steps, suitable premises, storage, transport, qualified and trained production and quality control personnel, adequate laboratory facilities, approved written procedures and instructions, records to show all steps of defined procedures taken, full traceability of a product through batch processing and distribution records, and systems for recall and investigation of complaints. The European Union's GMP (EU-GMP) guidelines implement similar requirements to those of the W.H.O. (C.E.C, 1992). The principles described above are general, and should be applied as governing principles during design, peer-review and evaluation of the ethical aspects of any clinical trial.

### United states (USA) regulatory issues

In the USA, MSCs are considered human cells, tissues, or cellular and tissue-based products (HCT/Ps), as specified in the definitions of The Code of Federal Regulation (CFR) Title 21 §1271 (21 CFR 1271). In 1993, the US FDA began establishing regulatory and guidance documentation for cell therapies with the issuance of the Application of Current Statutory Authority to Human Somatic Cell-therapy and Gene-therapy Products (F.D.A., 1993), which provided a biologics regulatory framework for the use of HCT/Ps. The tiered, risk-based approach means that products that present a lower perceived risk will be less regulated, while products with a larger perceived risk will undergo more extensive controls and examination. Both will require the cell products to be manufactured following Good Manufacturing Practices (GMP), and Good Tissue Practices (GTP). Additional regulatory requirements will depend on whether the cell product is “minimally manipulated” or “more-than-minimally manipulated.”

According to 21 CFR 1271.10, minimal manipulation criteria require that:

1. The HCT/P is minimally manipulated;
2. The HCT/P is intended for homologous use only, as reflected by the labeling, advertising, or other indications of the manufacturer's objective intent;
3. The manufacture of the HCT/P does not combine the cells or tissues with another article, except for water, crystalloids, or a sterilizing, preserving, or storage agent, provided that the addition of water, crystalloids, or the sterilizing, preserving, or storage agent does not raise new clinical safety concerns with respect to the HCT/P; and
4. Either:
  (4.1). The HCT/P does not have a systemic effect and is not dependent upon the metabolic activity of living cells for its primary function; or
  (4.2) The HCT/P has a systemic effect or is dependent upon the metabolic activity of living cells for its primary function, and: (a) is for autologous use; (b) is for allogeneic use in a first-degree or second-degree blood relative; or (c) is for reproductive use.

Minimal manipulation means processing that does not alter the relevant biological characteristics of cells or tissues. HCT/Ps that do not meet these four major criteria, are considered more-than-minimally-manipulated HCT/Ps. The FDA has stated that density-gradient separation, cell selection, centrifugation, and cryopreservation constitute minimal manipulation. All processes that manipulate the cell/tissue product, such as cell activation, encapsulation, *ex vivo* expansion, and gene modifications, are considered more-than-minimal manipulations. This will mostly be the case in many clinical applications of MSCs.

## Risk factor evaluation in applying MSC expanded cells

The principle of “ensure the safest possible method” should be applied when evaluating the feasibility of MSC-treatments in patients. When carrying out clinical trials using MSCs, there are some existing problems regarding the correct definition of risks related to the *in vitro* protocols used to prepare cells. In fact, the general types of risks reported for a CBMP should also be reported for MSC preparations. Specifically, human MSCs are living cells that are usually prepared by extensive culture in minimal essential media that contains supplements and reagents of animal origin, such as fetal calf serum and bovine trypsin. Thus, alongside the *infective risk* due to cell manipulation, patients treated with these cell preparations also face *prion exposure risk* due to the animal origin of the supplements, *toxicological risk* due to the persistence of toxic agents such as endotoxin, and *immunological risk* in case of contaminated proteins, peptides or other biomolecules of animal origin that could persist after cell harvesting and transplantation (Herberts et al., 2011). GMP procedures are intended to eliminate all of these risks and avoid exogenous contamination of the CBMP, while screening the final product by assaying the absence of various morbidity agents, such as microbial pathogens or endotoxins, deterministically assure the safety of the cell preparation in terms of infective, immunological and toxicological hazards.

Nonetheless, there are further risks related to the application of cultured MSCs in clinics, and the fact that risks are exclusively related to the biology of the cell themselves. In this context, the application of GMP procedures has no effect on the potential morbidity of the CBMP. The clinical appeal of MSCs is based on their high proliferative and differentiative potential, their ability to secrete cytokines/growth factors and their immunosuppressive function. However, these potentialities could also expose patients to *oncogenic/tumor-supporting risk* as well as to the *ectopic differentiation risk* (Herberts et al., 2011). There are increasing evidences that transplantation of MSCs in humans is safe in terms of transformation risk. Nonetheless, recent studies reported direct or indirect MSC-related tumorigenesis, in animals (Lepperdinger et al., 2008; Momin et al., 2010; Otto and Wright, 2011). A fundamental, still debated question is whether expansion of MSCs could lead to the acquisition of genomic abnormalities and consequently undergo oncogenic transformation. Recent investigations suggest that an abnormal karyotype not necessarily account for tumorigenic transformation but could be correlated to cell senescence. (Tarte et al., 2010; Sensebé, 2013; Wang et al., 2013). However, cell senescence could be related not only to the risk of transformation but also to the risk of inefficacy and increase in side effects.

The debated controversies on many aspects of MSC biology and the application of PP could represent a difficult obstacle to overcome. In order to facilitate the successful development of feasible CBMPs, the scientific community has put in great effort to get more information on the biological mechanism of MSCs, as well as precise characterization of the cells and reproducible production of the preparation batches. Nonetheless, is widely accepted that highly heterogeneous and different populations can be described under the generic terminology of MSCs (Ho et al., 2008). The question to be asked, therefore, is how it can be possible to evaluate risk factors applied to cells that are still far too ambiguous to be defined. Efficient prediction of the safety of MSC-based CBMP can be achieved deterministically for risks concerning exogenous contaminations by applying GMP procedures. On the contrary, precise evaluation of the intrinsic hazardousness related to the MSC biology should be done using a stochastic approach. In fact, sample harvesting, isolation, culture expansion, transport and administration of MSCs are greatly affected by an intractably large number of variables. In particular, emerging concepts such as “*multiple ancestors”* and “*culturing stochastic events*” should be taken into consideration, and attempts should be made to develop new and more efficient MSC-based therapeutic strategies.

Moreover, prediction of the beneficial effects of MSC-based therapy is exclusively correlated to the biology of the expanded cells, and the heterogeneity of the cell preparations could significantly affects the proliferative/differentiative potential, and consequently the regenerative potential, as well as immunoregolatory ability of the CBMP.

## *In vivo* multiple ancestors of MSCs and the deterministic model

From the very first evidence of the clinical value of the MSC-based therapies, it was clear that the definition provided by International Society for Cellular Therapy (ISCT) was not sufficiently stringent to determine a unique and unambiguous cell population. Different laboratories applied very different isolation protocols, and consequently produced significantly different cell populations (Wagner and Ho, 2007). These various protocols lead to expanded MSCs that differed greatly in terms of morphology, phenotype, proliferation and differentiation ability (Phinney, 2012). Nonetheless, many pre-clinical and clinical studies were conducted by applying this variety of cell products that were all defined under the widely accepted definition of MSCs, as a result of the non-stringent criteria of definition. Thus, the mechanism at the basis of MSC heterogeneity has usually been correlated not only to biological variability of donors, but also to a large number of variables, including tissue of origin, cell isolation and obviously, cell culture protocols (Phinney et al., 1999; Sharma et al., 2014).

While is widely accepted that the successful harvesting of MSCs, as well as their functionality, is highly affected by sex (Crisostomo et al., 2007; Deasy et al., 2007), age (Siegel et al., 2013), disease (Corre et al., 2007; Chen et al., 2012) and the pharmacological treatment (Lin et al., 2014) of donors, controversies about influence of tissue origins are still debated (Keating, 2012; Zhao, 2013). Emerging concepts about the tissue origins of MSCs suggest that MSCs are virtually present in all organs and tissue as a consequence of their perivascular localization, and that mild differences can be detected between expanded MSCs that are isolated from different organs under the same isolating and culture conditions (da Silva Meirelles et al., 2006). These differences may have been related to the influence of a modified local environment (niche) present in a different site of the body. Nonetheless, MSC isolating protocols usually include different steps in order to obtain a single cell suspension to be subsequently cultured. These isolating procedures could significantly differ in relation to the typology of tissue from which the MSCs should be extracted. (Pittenger et al., 1999; De Bari et al., 2001; Zuk et al., 2001; Alessandri et al., 2004; Bi et al., 2007; Barachini et al., 2009). Recently, a great influence of cell harvesting protocols as enzymatic digestion, centrifugation and other enrichment methods has been hypothesized (Shoshani et al., 2014). Thus, should this definitely demonstrate that MSCs reside in the vascular network of virtually all organs with no difference in their biology, as suggested by da Silva et al., the heterogeneity of these cells might be explained by three possibilities:

1. There is a unique *in vivo* MSC ancestor that is affected by the microenvironment in which it resides,
2. There is a unique *in vivo* MSC ancestor that is highly affected by the isolating procedures,
  or
3. There are multiple *in vivo* MSC ancestors that vary in proliferation and differentiation ability, which can be isolated in different portions in relation to the procedure applied.

In the last two decades, collecting evidences account for this third chance. From the seminal findings that MSCs could be isolated from the walls of blood vessels (Doherty et al., 1998; Bianco et al., 2001), strong evidence indicates that the fibroblastoid colonies originally described by Friedenstein (Friedenstein et al., 1968) originate from the *adventitia* of bone marrow sinusoids (Sacchetti et al., 2007). Successively, Tormin et al. demonstrated that *in vivo* MSC progenitors are not only present in the sub-endothelial layer of sinusoids but also resides in the trabecular bone-lining cell population (Tormin et al., 2011). Therefore, there are at least two cells able to generate MSCs from the bone marrow: one in the perivascular and one in the endosteal niche. Moreover, our group recently hypothesized that also lumen-facing layer of bone-marrow vessels and microvessels could host MSC progenitor, indicating a possible further MSC ancestors (Pacini and Petrini, 2014).

Similarly, adipose-derived MSCs, have been isolated from two distinct cell populations represented by pericytes encircling capillaries and microvessels, and adventitial cells surrounding larger arteries and veins (Corselli et al., 2012). MSC-like cultures can be expanded from both these populations, suggesting a dual vascular origin for the MSCs of the adipose tissue, similarly to what has been observed in bone marrow.

The relationship between these different progenitors remains obscure, despite that they have been largely characterized, and that many efforts have been made to identify the unique putative *in vivo* progenitors of MSCs from the different tissues (Bühring et al., 2007; Mabuchi et al., 2013).

Most of these studies include the application of immunological positive or negative selection of a specific sub-population, on the basis of a particular marker expression. This significantly contributes to increased confusion and controversies on the real identity of the MSC ancestors. In fact, a large number of antibody panels have been described as feasible for prospective isolation of MSCs, and various different sub-populations have been cultured to give rise to standard MSC cultures that match the ISCT criteria (Dominici et al., 2006). Interestingly, many of the identified *ex vivo* progenitors that are able to generate a standard MSC culture are represented by distinct populations. Thus, the search for the MSC ancestor is turning into a search for various MSC ancestors.

MSCs are commonly isolated from long-term cultures. It therefore, remains difficult to determine the primary cells of origin. The loose ISCT criteria hamper the identification of unique precursors of MSCs. Indeed, there are several types of primary cells with different features that can fulfill the definition of MSCs *in vitro*. Because the definition is not stringent, the presence of a unique common precursor for cells with MSC features cannot be hypothesized. In BM, MSCs can originate from both perivascular and endosteal progenitors; therefore, it is difficult to distinguish whether there is a unique common precursor, or if the loose ISCT definition is unable to identify two different progenitor populations. However, the clinical applications of MSCs are only partially limited by the incomplete characterization of the progenitor cells.

## Heterogeneity of culture expanded MSCs and the stochastic model

From the beginning of the MSC research, different terms have described the morphology of these plastic-adherent cells (Werts et al., 1980; Allen and Dexter, 1983; Kuznetsov et al., 1997; Colter et al., 2001), together with absence of specific surface markers to define expanded MSCs, the heterogeneous nature of this cell population lead to great confusion, made comparison from different studies problematic. Therefore, the differentiation toward seems to be the more reliable and stringent criteria to define MSCs. However, variability in the MSC differentiation potential has been observed between different sources (Strioga et al., 2012; Hoogduijn et al., 2014), different donors (Phinney et al., 1999), and also within different colonies obtained from the same subject (Russell et al., 2010), and also within the same colony (Digirolamo et al., 1999; Ylöstalo et al., 2008; Sengers et al., 2010). Thus, the minimum criteria defined early (Horwitz et al., 2005; Dominici et al., 2006) may now be simplistic and unnecessary (Keating, 2012).

Therefore, the term “multipotent mesenchymal stromal cells” does not identify a population of cultured cells with uniform features and unambiguous potential, but refers to a highly heterogeneous population that is dramatically affected by donor characteristics (Russell et al., 2013), isolation methods (Wagner and Ho, 2007; Barachini et al., 2009), media supplementation, seeding density, number of passages and culture time (Muraglia et al., 2000; Wagner and Ho, 2007; Barachini et al., 2009; Bieback et al., 2009; Shoshani et al., 2014).

One of the hypotheses about the reason of this variability takes in account the effects of stochastic events, which possibly occur during expansion (Pevsner-Fischer et al., 2011).

As discussed above, most of the clinical appeal of MSCs is represented by the fact that these cells are multipotent progenitors. This imply that MSCs, *in vitro* or *in vivo*, retain the ability to expand maintaining their features (self-renewal) or to generate vary cell populations committed toward some specific cell lineages (differentiation) (Mundra et al., 2013). All the actual und future MSC-based therapies are based on the assumption that administered MSC are going to meet a specific fate, in which their could differentiate (i.e., in tissue regeneration) or exert biological functions maintaining their biology (i.e., immunregulation and growth factor production) (Dimarino et al., 2013; Laroni et al., 2013; Bronckaers et al., 2014). In the recent years, many efforts have been applied in order to elucidate how primary stem as well as multipotent progenitor cells “decide” their fate. This represents a pivotal issue in studying mechanisms leading to tissue development, maintenance and consequently in approaching new MSC-based therapies (Thirumala et al., 2013).

Pluripotent cells reveal heterogeneous gene expression when exposed to a constant culture conditions. Recent technical developments in quantitative single-cell gene expression analysis (Larson et al., 2009; Raj and van Oudenaarden, 2009) have produces interesting results suggesting that individual cells can exhibit transient biases toward different lineages, even in clonal populations (Hough et al., 2009; Canham et al., 2010; Glotzbach et al., 2011; MacArthur et al., 2012; Moignard et al., 2013) as a results of this heterogeneity of gene expression, which might have a functional role in cell fate decisions. It has also been suggested that this molecular heterogeneity may result from stochastic fluctuations caused by noisy gene expression (Raser and O'Shea, 2005), leading to fluctuations in individual mRNA transcription and degradation rates, and in a similar manner for protein production, in individual cells (Elowitz et al., 2002; Ozbudak et al., 2002). These random repression/induction wavering in single-cell gene expression, predispose pluripotent cells to respond to specific environmental cues leading to differentiation (MacArthur and Lemischka, 2013). According to this model, development can be considered an intrinsically noisy system due to fluctuations in transcriptional regulation. These fluctuations in gene expression, that stochastically control cell fate, can be driven by intrinsic and extrinsic factors. Intrinsic factors result from randomness inherent to transcription and/or translation, while extrinsic factors derived from environmental fluctuations (Torres-Padilla and Chambers, 2014).

Stochastic models explaining gene expression heterogeneity have been also proposed *in vitro* for the embryonic stem cells (ESCs) (Wu and Tzanakakis, 2012), and extended to adult multipotent progenitor in lineage commitment during hemopoiesis (Teles et al., 2013). More specifically to MSCs, this model has been also proposed to account for the plasticity observed in these cells (Dennis and Charbord, 2002) and recently confirmed in the cardiomyogenic commitment of bone marrow-derived MSCs (Yamada et al., 2007).

**Figure 2:**
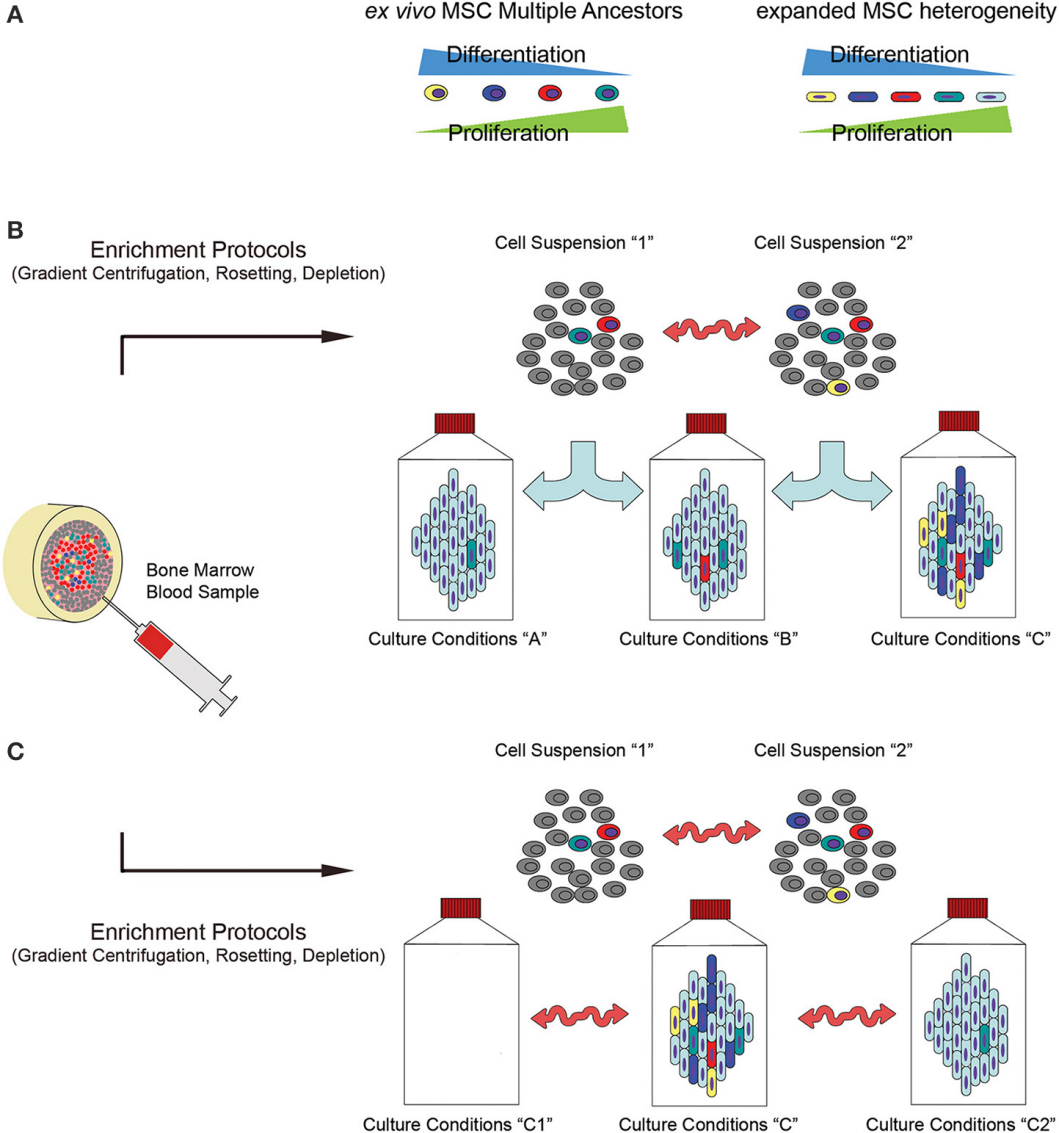
**Influence of culture determinants and stochastic events on the biology of bone-marrow-derived MSCs. (A)** Recent hypotheses suggest that MSCs may be derived from different progenitors that vary in terms of proliferative/differentiative ability and functionality, thus contributing to a high level of heterogeneity detected among the cell populations in the final products. Due to the presence of multiple *ex vivo* MSC progenitors, **(B)** different enrichment methods donor variability could lead to significantly different cell populations in terms of percentage of these various ancestors (indicated as “Cell Suspension 1” and “Cell Suspension 2”). Thus, applying different culture protocols (indicated as “Culture conditions A, B or C”) could select or simply promote specific subpopulations with different potential. **(C)** In addiction, even applying defined protocols, fluctuations in the environmental parameters could result in unpredictable differences in the culture conditions (indicated as “Culture conditions C, C1 or C2”) that lastly produce variability in the efficiency of the cell production process, and in the biology of the final product (proliferation, differentiation potential, immunoregolatory functions and growth factors/cytokine production).

Thus, not only specific culture conditions could select, or simply promote, particular subpopulations of MSCs, but in parallel environmental fluctuations could represent a stochastic proliferative advantage for individual cells. The hierarchical deterministic and stochastic model models possibly contribute to explain the observed morpho-functional variability of MSCs both *in vivo*, accounting for their multiple origins, and *in vitro* generating the heterogeneity of expanded cells (Figure 2).

## Deterministic and stochastic approaches in the evaluation of CBMP feasibility

Numerous multipotent cell populations has been isolated and expanded from various tissues, with some of them demonstrated the ability to differentiate not only toward the mesenchymal lineages but also toward other mesodermal cells (Sarugaser et al., 2009; Trombi et al., 2009). Moreover, MSC-like cells have been reported to be able to differentiate also into lineages from the three germ layers: endoderm, mesoderm and ectoderm (triploblastic differentiation) (Phinney and Prockop, 2007). However, the isolation and successive characterization of these subpopulations are strictly dependent on the application of specific culture condition, as well as specific purified culture-initiating cells (D'Ippolito et al., 2006; Kuroda et al., 2013).

Thus, either mild modifications in the culture determinants (culture-initiating cells and expansion protocols) or the environmental fluctuations during the cell manufacturing processes could affect the self-renewal/differentiation potential of the final cell product (Figure 2A), it is reasonable to suppose that a risks/benefits evaluation of CBMP could be strongly invalidated not only by different cell preparation protocols applied, but also by stochastic events that could take place during this phase. Different cell manufacturing processes may lead to significantly different cell products to be applied in CBMP preparation (Figure 2), depending on “macro-differences” in the starting material (tissue origins, isolating methods) and the cell production processes (media and supplements, pale blue arrows in Figure 2B), and “micro-differences” carried out by unpredictable parameter fluctuations during sampling (donor-to-donor variability, donor conditions at the time of sampling, red arrows in Figure 2B). Moreover, according to stochastic model, environmental parameter fluctuations could give rise to high level of heterogeneity, even if a defined expansion protocol was applied (red arrows in Figure 2C).

The possibility to predict the future success of a new MSC-based therapy is based on the assumption that the cell population applied in the *in vitro* and *in vivo* pre-clinical studies could be considerate equivalent to that applied in clinical trials. Noteworthy, clinical grade MSC production frequently forced to alter methods and reagents applied to fulfill GMP requirements (Fekete et al., 2012). Heterogeneity introduced by macro-differences could be almost eliminated applying predetermined clinical grade conditions also to the production of the cells designated for pre-clinical testing. Conversely, the micro-differences could only be phased out with a stochastic approach, where a particular level of uncertainty could affect a risk/benefits evaluation. The probability that a produced CBMP could result in an unexpected effect in treated patients is in tight correlation with the precise control of as many variables as possible during the all phases of the cell production (**Figure 4**, red boxes), and the following CBMP manufacture (**Figure 4**, black box).

### Tissue origin

In contrast to reported evidence that tissue origins could only mildly affect the MSC biology (da Silva Meirelles et al., 2006), consolidated evidence shows that multipotent MSCs from different sources (i.e., bone marrow, adipose tissue, Worton jelly) show differences in immunophenotype, proliferation and multilineage differentiation potential (Ishige et al., 2009; Strioga et al., 2012). Further investigations are needed to elucidate whether these modifications are introduced by the niche interactions or by the isolating procedures. In fact, in order to establish safe procedures for MSC-based CBMP productions, it is mandatory to set different guidelines for MSCs derived from any different source, and to standardize nomenclature that includes the origin of primary cultures, for instance: *bone marrow-derived mesenchymal stromal cells* (BM-MSCs) or *adipose tissue-derived MSCs* (AD-MSCs). Any search for a general consensus about risk exposure and beneficial effects of general MSCs that is done independently from the tissue origins risks failing.

### Isolating methods

**Figure 3:**
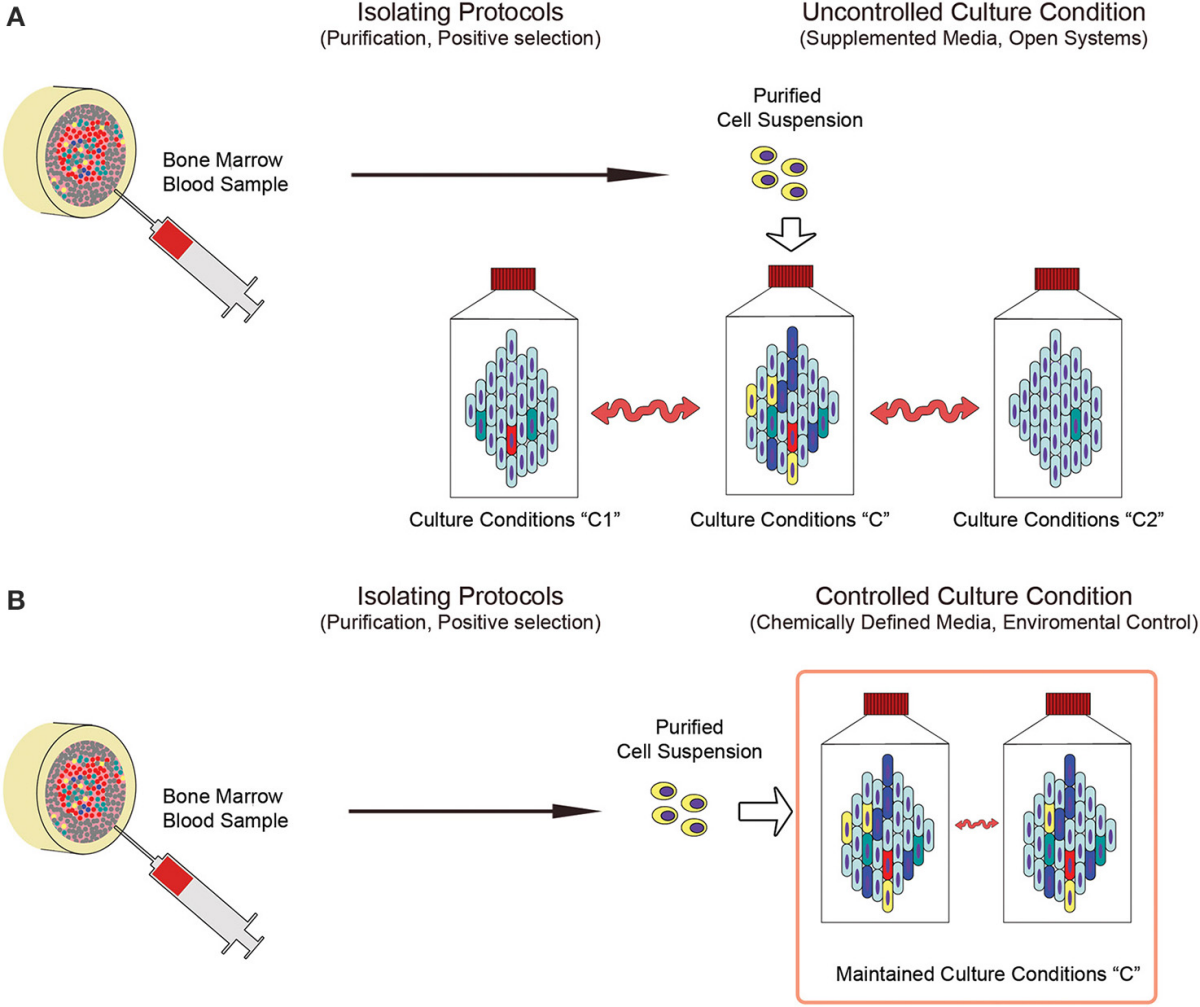
**Deterministic and stochastic approaches in the production of MSCs. (A)** Applying predetermined purified cell populations could drastically reduce the variability of starting material (indicated as “Purified Cell Suspension”) enabling the design of defined culture conditions that are specific to the unique culture-initiating cell population of interest. Nonetheless, applying uncontrolled culture systems do not prevent from the heterogeneity introduced by environmental parameter fluctuations, resulting in mild, but significant, differences in the culture conditions (indicated as “Culture conditions C, C1 or C2”). **(B)** Conversely, close culture systems under constant environmental parameters control, in association with chemically defined media, could represent feasible tools limiting fluctuations (indicated as “Maintained Culture Condition C”) and increasing reproducibility of the cell products.

Using single cell suspension as starting material is usually required to initiate an expanding MSC culture protocol. The methodologies applied to obtain it include tissue digestion, cell enrichment (i.e., density gradient centrifugation or rosetting) or even direct isolation of putative *in vivo* precursors on the basis of immunophenotypic features (i.e., MSCA-1, CD271, STRO-1 or SSEA-4) (Gang et al., 2007; Psaltis et al., 2010), adhesion properties (i.e., MPCs) (Trombi et al., 2009; Fazzi et al., 2011) or morphological features (i.e., VSEL cells) (Ratajczak et al., 2008). In order to optimize isolating procedures, it would be useful to identify the most advantageous methods in terms of quality and quantity of isolated cells, MSC-colony forming unit frequency (CFU-MSCs), ease of performing the process, safety in terms of exposition to exogenous contamination, and costs (Hideki, 2013). Nonetheless, due to the possible multiple origins of MSCs (Figure 2A) discussed above and the alterations of the cell composition induced by the *ex vivo* procedures (Figures 2B,C), highly purified cell populations are preferable as MCS culture-initiating cells. A probabilistic prediction of risks and benefits would be more accurate in reducing biological variability of starting materials. Highly purified initiating cells would also limit variability induced by sex, age, diseases and pharmacological treatments of donors at the time of sampling, that could alter the composition of simply enriched cell suspensions. However, environmental fluctuations could still introduce certain level of heterogeneity of the final product, even if in a lesser extent (Figure 3A).

### Culture systems

One of the most important factors affecting heterogeneity of cultured MSC is introduced by serum supplementation (Bieback et al., 2009). Even if it were possible to standardize origins (bovine or human) or the screening quality of sera, batch-to-batch variability is related to the unpredictable number of influencing factors that avoid controlling the concentration of growth factors, cytokines, hormones and other biomolecules in the culture media. Similarly, other supplements, such as bovine pituitary gland or human platelet lysates were affected by the same uncertainty. In order to accurately predict the feasibility of MSC-based treatments, any batch-to-batch variability should be eliminated or drastically reduced. In fact, even if highly purified cell populations are used as starting materials, it is still possible to obtain different CBMPs after culturing in media that may differ in some or many biomolecules with potent effects on MSC biology (Figure 3A).

Chemically defined and xeno-free media (CDM) should be used in order to more precisely evaluate the clinical value of expanded MSCs. CDM can be produced under strictly defined quality control requirements to reduce batch variability and to guarantee a higher level of reproducibility in the CBMP production.

Nonetheless, many other culture parameters can affect the final cell product, such as cell density, time of culture, number of passages, frequency of media change, temperature fluctuations, and pH and oxygen tension alterations (D'Ippolito et al., 2006). In addition, the process of culturing MSCs in open systems, even if conducted under stringent GMP requirements, may be affected by the frequent alteration of environmental parameters such as temperature, P_CO2_, P_O2_, pH and humidity that regulates evaporation/condensation of a medium due to the culture maintenance. In fact, the discontinuous medium change includes the need for moving culture flasks from the incubator to the cabinet and vice versa. Processing a large number of flasks and/or large volumes of medium could result in a significant gap of time from the first flask processed to the last one, exposing cultures to unsuitable environmental parameters. Moreover, open systems require significant manipulation by operators, which exposes the CBMP production to unpredictable human error. Thus, in the view of reducing variability in the production process in order to increase feasibility predictions, employing dedicated bioreactors (i.e., hollow-fibers culture systems) should be mandatory. In these closed computerized systems, it would be possible to automate media changes, or even eliminate them, by constant flow of fresh medium, keeping cultures under proper incubating conditions and reducing operator involvement. Additionally, it is important that bioreactors that are employed to produce clinical grade MSCs are equipped with sensors that monitor culture parameters such as osmolarity, pH or oxygen tension, and can produce data-logs of the entire culture process. These apparatuses, especially hollow-fiber culture systems, could also have two other significant advantages: (1) producing clinical-grade MSCs in a “closed system,” drastically reducing the risk of exogenous contamination due to the limited culture manipulation, and (2) provide vast culture surfaces in relative small volumes, making cell passaging (sub-cultures) unnecessary. The disadvantage in applying computerized bioreactors, alongside the significant increase in cost, lies in the shear stress introduced by the exposition of the cells to a constant liquid flow. Shear stress has been reported to significantly affect proliferation and differentiation potential of MSCs (McCoy and O'Brien, 2010; Yeatts and Fisher, 2011), however this could be easily monitored and controlled in a computer-assisted culture system, making this parameter also suitable to intentionally induce particular cell behavior (Yeatts et al., 2013). Thus, application of CDM in conjunction with environmental controlled culture systems could significantly reduce culture condition fluctuations, increasing reproducibility of the cell products (Figure 3B).

### Final product release

Quality control (QC) requirements of the final cell preparations should be indicated and standardized during the pre-clinical phase. Alongside microbiological, endotoxin and pyrogenicity testing, the releasing QCs should include an evaluation of the number, vitality and senescence of cells, which could affect the efficacy of the treatment and determination of the minimal dose. Phenotypes could be also investigated, but the current lack of specific and widely accepted MSC markers make the immuno-phenotypization of the CBMP barely determinant in view of comparative studies. ISCT nomenclature should be refined, and in addition to standard methods of cell characterization, as surface marker profile and differentiation potential assays, more advanced molecular tools including assessments of the cell transcriptome, proteome, and secretome should be evaluated in creating effective and discriminating QC requirements (Keating, 2012; Ranganath et al., 2012). Moreover, genetic stability of the cell product should assay, even if a definitive correlation to tumorigenicity is still lacking. Conventional karyotyping has been suggested to be not sufficient to predict full genome stability, due to the poor sensitivity and resolution of this test (Barkholt et al., 2013). Thus, release tests should include genome analysis with higher performances as comparative genomic hybridization array (CGH array). Fluorescent *in situ* hybridization (FISH) analysis could be also applied to detect recurrent abnormalities that possibly have been previously associated to long-term cultured MSCs (Wang et al., 2013).

However, the release of the final product should be primarily dependent upon the validation of the entire production process. In fact, the ability to efficiently predict the CBMP response in humans is correlated to the possibility of assimilating the CBMP itself to the products evaluated in the pre-clinical tests. Thus, each phase of the cell preparation should be validated by pre-determinate requirements, such as the quality of the starting material (i.e., hemodilution of bone marrow samples), the quality of the isolating cell population (purity and vitality assay) and the culture process (data-logs validation).

### Cell product freezing and storage

Cryopreservation and storage of clinical-grade MSCs offers unique opportunities to advance the potential uses of these cells, allowing cell banking, transportation, cell product back-up and repeated infusions. In clinical banking of MSCs, special consideration should be taken in consideration applying freezing process. (Thirumala et al., 2013; Sharma et al., 2014). In fact, crypreservation could be assimilated to the culturing expansion process where crypreservation media, supplements (i.e., animal sera) and additives (i.e., DMSO) could represent a further cause of exposition to the infective, prion, toxicological and immunological risks discussed above. Moreover, crypreservation, similarly to cell expansion, is a process highly sensitive to environmental fluctuations. Thus, consistent and reproducible rates of temperature change during freezing and thawing are mandatory and could be only achieved applying automated and controlled devices. In the same manner the necessary removal of DMSO, as other additive, should be conducted in close cell washing systems, in order to maintain GMP compliance. Moreover, constant temperature control should be assured during long-term storage in pharmaceutical grade liquid nitrogen (Liras, 2010; Thirumala et al., 2013).

### Application of MSC-based CBMP

Most of the biological features and functions of expanded MSC can be investigated by applying protocols that may take one to several weeks to be evaluated. Many of these features are very important for applying MSC in clinics, and include tumorigeneity, tumor support, differentiation potential and immunoregulatory functions. Hence, it is not possible to evaluate these very important biological features at the time of the CBMP release, and pre-clinical studies are mandatory.

The objectives of the pre-clinical studies are to provide a proof-of-principle demonstration of the safety and efficacy of a CBMP, and to define the pharmacological and toxicological effects that are predictive of the responses in humans. Moreover, there are several “post-production” factors that are not strictly correlated to the biology of the CBMP, but that could be influenced by it. These include administration, supporting pharmacological treatments and follow-up of treated patients. Pre-clinical studies also allow for the establishment of safe and effective doses, the investigation of routes and methods of CBMP administration and the identification of target organs for possible toxicity, which enable the definition of parameters to be monitored in the patients. In order to obtain a predictive proof-of-principle demonstration of a CBMP-based therapy, pre-clinical studies should be performed in relevant animal models, meaning that the animals should allow the human response to the CBMP to be predicted. However, for certain preclinical studies, such as the biodistribution of cells, a homologous model might provide more valuable information than xenogeneic models, such as human cells in immune-suppressed or immunodeficient animals.

## Conclusions

**Figure 4:**
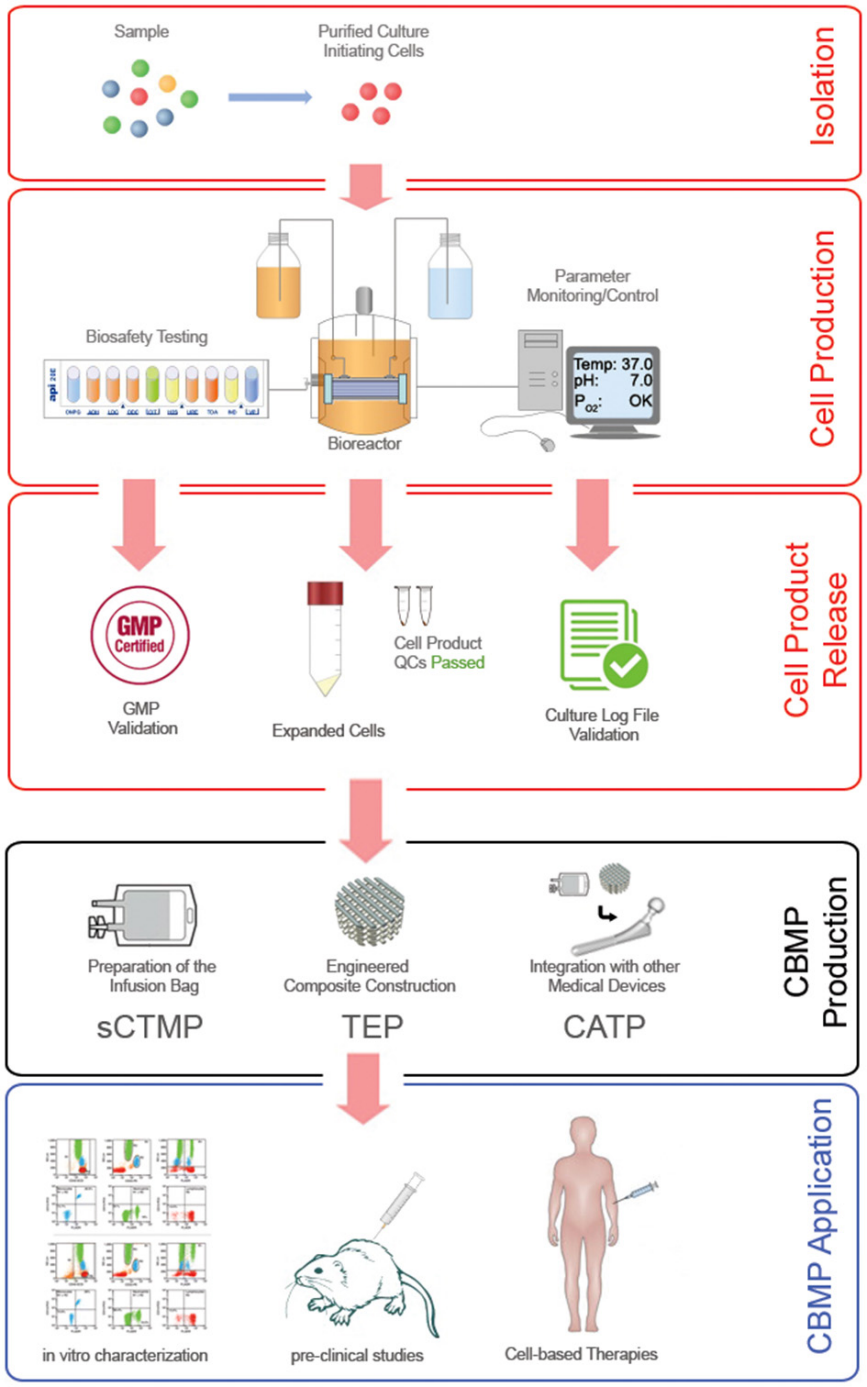
**Ideal MSC-based CBMP production process**. Robust reproducibility in the MSC expansion/production process (red frames) can be achieved by applying specific isolating methods that are able to supply defined culture-initiating cells, and highly controlled cell expansion cultures conducted in computerized close systems. Final product release should be subjected not only to GMP validation to ensure the bio-safety of the cell product, but also to quality control validation of the entire production process. Monitoring and control of environmental parameters can significantly increase the predictability of the *in vitro* characterizations and pre-clinical studies. In fact, drastically reducing the batch-to-batch heterogeneity of expanded MSCs and consequently of the CBMP produced (black frame) for different applications (blue frame) could result in less variation in the clinical outcome, and lastly improve the efficacy of cell-based therapies.

Although MSCs might be considered the most intensely studied multipotent cell types in cell-based therapy, comparison of existing pre-clinical and clinical data reveal that the risk/benefit analysis of an MSC-based treatment may be affected by a significant level of uncertainty. The reduced predictability of pre-clinical studies limits the CBMP feasibility required by PP. Standardization and harmonization of protocols concerning tissue origins, isolating methods, expansion culture, characterization and quality controls are still lacking. Thus, in recent years many efforts have been made to establish optimized protocols for GMP-compliant preparation of MSCs, develop tools and a catalog of markers for their characterization, and define their differentiation potential *in vitro* and in animal models. Nonetheless, recent hypotheses suggest that a widely accepted consensus about MSC preparation could be an out-of-reach goal. Heterogeneity of MSC *ex vivo* progenitors, as well as variability introduced by cell manipulation, do not allow deterministic definition of unique CBMP producing protocol. Culturing MSCs is not a static procedure, and it is highly sensible to environmental clues. MSCs are living production factories on their own that react to environmental clues, *in vivo* and *in vitro*, and in response change their behavior. Using a robust and reproducible production regime for these cells is of high necessity, as is the development of standardized bioreactors (Figure 4, red boxes). Applying purified cell populations as culture-initiating cells under specific and finely controlled culture conditions could strongly increase the reproducibility of the released CBMPs (Figure 4, black box) the predictability of the pre-clinical studies regarding for a particular clinical application, and could result in less variation in the outcome, as well as boost future clinical applications (Figure 4, blue box).

### Conflict of interest statement

The author declares that the research was conducted in the absence of any commercial or financial relationships that could be construed as a potential conflict of interest.

## References

[B1] Alessandri G. Pagano S. Bez A. Benetti A. Pozzi S. Iannolo G.. (2004). Isolation and culture of human muscle-derived stem cells able to differentiate into myogenic and neurogenic cell lineages. Lancet 26, 1872–1883. 10.1016/S0140-6736(04)17443-6 15555667

[B2] Allen T. D. Dexter T. M. (1983). Long term bone marrow cultures: an ultrastructural review. Scan Electron. Microsc. (Pt 4), 1851–1866. 6669951

[B3] Allison M. (2009). Genzyme backs Osiris, despite Prochymal flop. Nat. Biotechnol. 27, 966–967. 10.1038/nbt1109-966 19898434

[B4] Au P. Tam J. Fukumura D. Jain R. K. (2008). Bone marrow-derived mesenchymal stem cells facilitate engineering of long-lasting functional vasculature. Blood 111, 4551–4558. 10.1182/blood-2007-10-118273 18256324 PMC2343592

[B5] Barachini S. Trombi L. Danti S. D'Alessandro D. Battolla B. Legitimo A.. (2009). Morpho-functional characterization of human mesenchymal stem cells from umbilical cord blood for potential uses in regenerative medicine. Stem Cells Dev. 18, 293–305. 10.1089/scd.2008.0017 18444788

[B6] Barkholt L. Flory E. Jekerle V. Lucas-Samuel S. Ahnert P. Bisset L.. (2013). Risk of tumorigenicity in mesenchymal stromal cell-based therapies—bridging scientific observations and regulatory viewpoints. Cytotherapy 15, 753–759. 10.1016/j.jcyt.2013.03.005 23602595

[B7] Bi Y. Ehirchiou D. Kilts T. M. Inkson C. A. Embree M. C. Sonoyama W.. (2007). Identification of tendon stem/progenitor cells and the role of the extracellular matrix in their niche. Nat. Med. 10, 1219–1227. 10.1038/nm1630 17828274

[B8] Bianco P. Riminucci M. Gronthos S. Robey P. G. (2001). Bone marrow stromal stem cells: nature, biology, and potential applications. Stem Cells 19, 180–192. 10.1634/stemcells.19-3-180 11359943

[B9] Bieback K. Hecker A. Kocaömer A. Lannert H. Schallmoser K. Strunk D.. (2009). Human alternatives to fetal bovine serum for the expansion of mesenchymal stromal cells from bone marrow. Stem Cells 27, 2331–2341. 10.1002/stem.139 19544413

[B10] Bronckaers A. Hilkens P. Martens W. Gervois P. Ratajczak J. Struys T.. (2014). Mesenchymal stem/stromal cells as a pharmacological and therapeutic approach to accelerate angiogenesis. Pharmacol. Ther. 143, 181–196. 10.1016/j.pharmthera.2014.02.013 24594234

[B11] Bühring H. J. Battula V. L. Treml S. Schewe B. Kanz L. Vogel W. (2007). Novel markers for the prospective isolation of human MSC. Ann. N.Y. Acad. Sci. 1106, 262–271 10.1196/annals.1392.000 17395729

[B12] Canham M. A. Sharov A. A. Ko M. S. Brickman J. M. (2010). Functional heterogeneity of embryonic stem cells revealed through translational amplification of an early endodermal transcript. PLoS Biol. 8:e1000379. 10.1002/jcb.24166 20520791 PMC2876051

[B13] Cao Y. Sun Z. Liao L. Meng Y. Han Q. Zhao R. C. (2005). Human adipose tissue derived stem cells differentiate into endothelial cells *in vitro* and improve postnatal neovascularization *in vivo*. Biochem. Biophys. Res. Commun. 332, 370–379. 10.1016/j.bbrc.2005.04.135 15896706

[B14] C.E.C. (Commission of the European Communities). (1992). Guide to Good Manufacturing Practice (GMP) for Medical Products. The Rules Governing Medicinal Products in the European Community, Vol IV

[B15] Chen H. T. Lee M. J. Chen C. H. Chuang S. C. Chang L. F. Ho M. L.. (2012). Proliferation and differentiation potential of human adipose-derived mesenchymal stem cells isolated from elderly patients with osteoporotic fractures. J. Cell. Mol. Med. 16, 582–593. 10.1111/j.1582-4934.2011.01335.x 21545685 PMC3822933

[B16] Colter D. C. Sekiya I. Prockop D. J. (2001). Identification of a subpopulation of rapidly self-renewing and multipotential adult stem cells in colonies of human marrow stromal cells. Proc. Natl. Acad. Sci. U.S.A. 98, 7841–7845. 10.1073/pnas.141221698 11427725 PMC35429

[B17] COM.EST (World Commission on the Ethics of Scientific Knowledge and Technology). (2005). The Precautionary Principle. Published in March 2005 by UNESCO.

[B18] Corre J. Mahtouk K. Attal M. Gadelorge M. Huynh A. Fleury-Cappellesso S.. (2007). Bone marrow mesenchymal stem cells are abnormal in multiple myeloma. Leukemia 21, 1079–1088. 10.1038/sj.leu.2404621 17344918 PMC2346535

[B19] Corselli M. Chen C. W. Sun B. Yap S. Rubin J. P. Péault B. (2012). The tunica adventitia of human arteries and veins as a source of mesenchymal stem cells. Stem Cells Dev. 21, 1299–1308. 10.1089/scd.2011.0200 21861688 PMC3353742

[B20] Crisostomo P. R. Wang M. Herring C. M. Markel T. A. Meldrum K. K. Lillemoe K. D.. (2007). Gender differences in injury induced mesenchymal stem cell apoptosis and VEGF, TNF, IL-6 expression: role of the 55 kDa TNF receptor (TNFR1). J. Mol. Cell. Cardiol. 42, 142–149. 10.1016/j.yjmcc.2006.09.016 17070836 PMC1779905

[B21] da Silva Meirelles L. Chagastelles P. C. Nardi N. B. (2006). Mesenchymal stem cells reside in virtually all post-natal organs and tissues. J. Cell Sci. 119, 2204–2211. 10.1242/jcs.02932 16684817

[B22] Deasy B. M. Lu A. Tebbets J. C. Feduska J. M. Schugar R. C. Pollett J. B.. (2007). A role for cell sex in stem cell-mediated skeletal muscle regeneration: female cells have higher muscle regeneration efficiency. J. Cell Biol. 177, 73–86. 10.1083/jcb.200612094 17420291 PMC2064113

[B23] De Bari C. Dell'Accio F. Tylzanowski P. Luyten F. P. (2001). Multipotent mesenchymal stem cells from adult human synovial membrane. Arthritis Rheum. 44, 1928–1942. 10.1002/1529-0131(200108)44:8<1928::AID-ART331>3.0.CO;2-P 11508446

[B24] Dennis J. E. Charbord P. (2002). Origin and differentiation of human and murine stroma. Stem Cells 20, 205–214. 10.1634/stemcells.20-3-205 12004079

[B25] Digirolamo C. M. Stokes D. Colter D. Phinney D. G. Class R. Prockop D. J. (1999). Propagation and senescence of human marrow stromal cells in culture: a simple colony-forming assay identifies samples with the greatest potential to propagate and differentiate. Br. J. Haematol. 107, 275–281. 10.1046/j.1365-2141.1999.01715.x 10583212

[B26] Dimarino A. M. Caplan A. I. Bonfield T. L. (2013). *Mesenchymal stem cells in tissue* repair. Front. Immunol. 4:201. 10.3389/fimmu.2013.00201 PMC3761350 24027567

[B27] D'Ippolito G. Howard G. A. Roos B. A. Schiller P. C. (2006). Isolation and characterization of marrow-isolated adult multilineage inducible (MIAMI) cells. Exp. Hematol. 34, 1608–1610. 10.1016/j.exphem.2006.07.016 17046585

[B28] Doherty M. J. Ashton B. A. Walsh S. Beresford J. N. Grant M. E. Canfield A. E. (1998). Vascular pericytes express osteogenic potential *in vitro* and *in vivo*. J. Bone Miner. Res. 13, 828–838. 10.1359/jbmr.1998.13.5.828 9610747

[B29] Dominici M. Le Blanc K. Mueller I. Slaper-Cortenbach I. Marini F. Krause D.. (2006). Minimal criteria for defining multipotent mesenchymal stromal cells. The international society for cellular therapy position statement. Cytotherapy 8, 315–317. 10.1080/14653240600855905 16923606

[B30] Elowitz M. B. Levine A. J. Siggia E. D. Swain P. S. (2002). Stochastic gene expression in a single cell. Science 297, 1183–1186. 10.1126/science.1070919 12183631

[B31] Fazzi R. Pacini S. Carnicelli V. Trombi L. Montali M. Lazzarini E.. (2011). Mesodermal progenitor cells (MPCs) differentiate into mesenchymal stromal cells (MSCs) by activation of Wnt5/calmodulin signalling pathway. PLoS ONE 6:e25600. 10.1371/journal.pone.0025600 21980498 PMC3183072

[B32] F.D.A. (US Food and Drug Administration). (1993). Application of current statutory authority to human somatic cell-therapy and gene-therapy products. Fed. Reg. 58, 53248–53251

[B33] Fekete N. Rojewski M. T. Fürst D. Kreja L. Ignatius A. Dausend J.. (2012). GMP-compliant isolation and large-scale expansion of bone marrow-derived MSC. PLoS ONE 7:e43255. 10.1371/journal.pone.0043255 22905242 PMC3419200

[B34] Friedenstein A. J. Petrakova K. V. Kurolesova A. I. Frolova G. P. (1968). Heterotopic of bone marrow. Analysis of precursor cells for osteogenic and hematopoietic tissues. Transplantation 6, 230–247. 10.1097/00007890-196803000-00009 5654088

[B35] Gálvez P. Clares B. Hmadcha A. Ruiz A. Soria B. (2013). Development of a cell-based medicinal product: regulatory structures in the European Union. Br. Med. Bull. 105, 85–105. 10.1093/bmb/lds036 23184855

[B111] Gang E. J. Bosnakovski D. Figueiredo C. A. Visser J. W. Perlingeiro R. C. (2007). SSEA-4 identifies mesenchymal stem cells from bone marrow. Blood 109, 1743–1751. 10.1182/blood-2005-11-010504 17062733

[B36] Glennie S. Soeiro I. Dyson P. J. Lam E. W. Dazzi F. (2005). Bone marrow mesenchymal stem cells induce division arrest anergy of activated T cells. Blood 105, 2821–2827. 10.1182/blood-2004-09-3696 15591115

[B37] Glotzbach J. P. Januszyk M. Vial I. N. Wong V. W. Gelbard A. Kalisky T.. (2011). An information theoretic, microfluidic-based single cell analysis permits identification of subpopulations among putatively homogeneous stem cells. PLoS ONE 6:e21211. 10.1371/journal.pone.0021211 21731674 PMC3120839

[B38] Herberts C. A. Kwa M. S. Hermsen H. P. (2011). Risk factors in the development of stem cell therapy. J. Transl. Med. 22:29. 10.1186/1479-5876-9-29 21418664 PMC3070641

[B39] Hideki A. (2013). Isolation of bone marrow stromal cells: cellular composition is technique-dependent, in Regenerative Medicine and Tissue Engineering, ed Andrades J. A. 37–50. 10.5772/55543

[B40] Ho A. D. Wagner W. Franke W. (2008). Heterogeneity of mesenchymal stromal cell preparations. Cytotherapy 10, 320–330. 10.1080/14653240802217011 18574765

[B41] Hoogduijn M. J. Betjes M. G. Baan C. C. (2014). Mesenchymal stromal cells for organ transplantation: different sources and unique characteristics? Curr. Opin. Organ Transplant. 19, 41–46. 10.1097/MOT.0000000000000036 24275893

[B42] Horwitz E. M. Andreef M. Frassoni F. (2006). Mesenchymal stromal cells. Curr. Opin. Hematol. 13, 419–425. 10.1097/01.moh.0000245697.54887.6f 17053453 PMC3365862

[B43] Horwitz E. M. Le Blanc K. Dominici M.. (2005). Clarification of the nomenclature for MSC: the International Society for Cellular Therapy position statement. Cytotherapy 7, 393–395. 10.1080/14653240500319234 16236628

[B44] Horwitz E. M. Prockop D. J. Fitzpatrick L. A. Koo W. W. Gordon P. L. Neel M.. (1999). Transplantability and therapeutic effects of bone marrow-derived mesenchymal cells in children with osteogenesis imperfecta. Nat. Med. 5, 309–313. 10.1038/6529 10086387

[B45] Hough S. R. Laslett A. L. Grimmond S. B. Kolle G. Pera M. F. (2009). A continuum of cell states spans pluripotency and lineage commitment in human embryonic stem cells. PLoS ONE 4:e7708. 10.1371/journal.pone.0007708 19890402 PMC2768791

[B46] Ishige I. Nagamura-Inoue T. Honda M. J. Harnprasopwat R. Kido M. Sugimoto M.. (2009). Comparison of mesenchymal stem cells derived from arterial, venous, and Wharton's jelly explants of human umbilical cord. Int. J. Hematol. 90, 261–269. 10.1007/s12185-009-0377-3 19657615

[B47] Keating A. (2012). Mesenchymal stromal cells: new directions. Cell Stem Cell 10, 709–716. 10.1016/j.stem.2012.05.015 22704511

[B48] Kuroda Y. Wakao S. Kitada M. Murakami T. Nojima M. Dezawa M. (2013). Isolation, culture and evaluation of multilineage-differentiating stress-enduring (Muse) cells. Nat. Protoc. 8, 1391–1415. 10.1038/nprot.2013.076 23787896

[B49] Kuznetsov S. A. Krebsbach P. H. Satomura K. Kerr J. Riminucci M. Benayahu D.. (1997). Single-colony derived strains of human marrow stromal fibroblasts form bone after transplantation *in vivo*. J. Bone Miner. Res. 12, 1335–1347. 10.1359/jbmr.1997.12.9.1335 9286749

[B50] Laroni A. Novi G. Kerlero de Rosbo N. Uccelli A. (2013). Towards clinical application of mesenchymal stem cells for treatment of neurological diseases of the central nervous system. J Neuroimmune Pharmacol. 8, 1062–1076. 10.1007/s11481-013-9456-6 23579931

[B51] Larson D. R. Singer R. H. Zenklusen D. (2009). A single molecule view of gene expression. Trends Cell Biol. 19, 630–637. 10.1016/j.tcb.2009.08.008 19819144 PMC2783999

[B52] Le Blanc K. Tammik C. Rosendahl K. Zetterberg E. Ringdén O. (2003). HLA expression and immunologic properties of differentiated and undifferentiated mesenchymal stem cells. Exp. Hematol. 31, 890–896. 10.1016/S0301-472X(03)00110-3 14550804

[B53] Lepperdinger G. Brunauer R. Jamnig A. Laschober G. Kassem M. (2008). Controversial issue: is it safe to employ mesenchymal stem cells in cell-based therapies? Exp. Gerontol. 43, 1018–1023. 10.1016/j.exger.2008.07.004 18694815

[B54] Lin H.-H. Hwang S.-M. Wu S.-J. Hsu L.-F. Liao Y.-H.. (2014). The osteoblastogenesis potential of adipose mesenchymal stem cells in myeloma patients who had received intensive therapy. PLoS ONE 9:e94395. 10.1371/journal.pone.0094395 24722177 PMC3983165

[B55] Liras A. (2010). Future research and therapeutic applications of human stem cells: general, regulatory, and bioethical aspects. J. Transl. Med. 8:131. 10.1186/1479-5876-8-131 21143967 PMC3014893

[B56] Mabuchi Y. Houlihan D. D. Akazawa C. Okano H. Matsuzaki Y. (2013). Prospective isolation of murine and human bone marrow mesenchymal stem cells based on surface markers. Stem Cells Int. 2013:507301. 10.1155/2013/507301 23766770 PMC3673454

[B57] MacArthur B. D. Sevilla A. Lenz M. Müller F. J. Schuldt B. M. Schuppert A. A.. (2012). Nanog-dependent feedback loops regulate murine embryonic stem cell heterogeneity. Nat. Cell Biol. 14, 1139–1147. 10.1038/ncb2603 23103910 PMC3507454

[B58] MacArthur B. D. Lemischka I. R. (2013). Statistical mechanics of pluripotency. Cell 154, 484–489. 10.1016/j.cell.2013.07.024 23911316

[B59] Malliaras K. Kreke M. Marbán E. (2011). The stuttering progress of cell therapy for heart disease. Clin. Pharmacol. Ther. 90, 532–541. 10.1038/clpt.2011.175 21900888

[B60] McCoy R. J. O'Brien F. J. (2010). Influence of shear stress in perfusion bioreactor cultures for the development of three-dimensional bone tissue constructs: a review. Tissue Eng. Part B Rev. 16, 587–601. 10.1089/ten.teb.2010.0370 20799909

[B61] Moignard V. Macaulay I. C. Swiers G. Buettner F. Schütte J. Calero-Nieto F. J.. (2013). Characterization of transcriptional networks in blood stem and progenitor cells using high-throughput single-cell gene expression analysis. Nat. Cell Biol. 15, 363–372. 10.1038/ncb2709 23524953 PMC3796878

[B62] Momin E. N. Vela G. Zaidi H. A. Quiñones-Hinojosa A. (2010). The oncogenic potential of mesenchymal stem cells in the treatment of cancer: directions for future research. Curr. Immunol. Rev. 6, 137–148. 10.2174/157339510791111718 20490366 PMC2873198

[B63] Muguruma Y. Yahata T. Miyatake H. Sato T. Uno T. Itoh J.. (2006). Reconstitution of the functional human hematopoietic microenvironment derived from human mesenchymal stem cells in the murine bone marrow compartment. Blood 107, 1878–1888. 10.1182/blood-2005-06-2211 16282345

[B64] Mundra V. Gerling I. C. Mahato R. I. (2013). Mesenchymal stem cell-based therapy. Mol. Pharm. 10, 77–89. 10.1021/mp3005148 23215004 PMC3549356

[B65] Muraglia A. Cancedda R. Quarto R. (2000). Clonal mesenchymal progenitors from human bone marrow differentiate *in vitro* according to a hierarchical model. J. Cell Sci. 113(Pt 7), 1161–1166. 10704367 10.1242/jcs.113.7.1161

[B66] Otto W. R. Wright N. A. (2011). Mesenchymal stem cells: from experiment to clinic. Fibrogenesis Tissue Repair. 8, 4–20. 10.1186/1755-1536-4-20 21902837 PMC3182886

[B67] Ozbudak E. M. Thattai M. Kurtser I. Grossman A. D. van Oudenaarden A. (2002). Regulation of noise in the expression of a single gene. Nat. Genet. 31, 69–73. 10.1038/ng869 11967532

[B68] Pacini S. Petrini I. (2014). Are MSCs angiogenic cells? New insights on human nestin-positive bone marrow-derived multipotent cells. Front. Cell Dev. Biol. 19:20. 10.3389/fcell.2014.00020 PMC4207020 25364727

[B69] Pevsner-Fischer M. Levin S. Zipori D. (2011). The origins of mesenchymal stromal cell heterogeneity. Stem Cell Rev. 7, 560–568. 10.1007/s12015-011-9229-7 21437576

[B70] Phinney D. G. (2012). Functional heterogeneity of mesenchymal stem cells: implications for cell therapy. J. Cell. Biochem. 113, 2806–2812. 10.1002/jcb.24166 22511358

[B71] Phinney D. G. Kopen G. Righter W. Webster S. Tremain N. Prockop D. J. (1999). Donor variation in the growth properties and osteogenic potential of human marrow stromal cells. J. Cell. Biochem. 75, 424–436. 10536366

[B72] Phinney D. G. Prockop D. J. (2007). Concise review: mesenchymal stem/multipotent stromal cells: the state of transdifferentiation and modes of tissue repair—current views. Stem Cells 25, 2896–2902. 10.1634/stemcells.2007-0637 17901396

[B73] Pittenger M. F. Mackay A. M. Beck S. C. Jaiswal R. K. Douglas R. Mosca J. D.. (1999). Multilineage potential of adult human mesenchymal stem cells. Science 284, 143–147. 10.1126/science.284.5411.143 10102814

[B74] Psaltis P. J. Paton S. See F. Arthur A. Martin S. Itescu S.. (2010). Enrichment for STRO-1 expression enhances the cardiovascular paracrine activity of human bone marrow-derived mesenchymal cell populations. J. Cell. Physiol. 223, 530–540. 10.1002/jcp.22081 20162565

[B75] Raj A. van Oudenaarden A. (2009). Single-molecule approaches to stochastic gene expression. Annu. Rev. Biophys. 38, 255–270. 10.1146/annurev.biophys.37.032807.125928 19416069 PMC3126657

[B76] Ranganath S. H. Levy O. Inamdar M. S. Karp J. M. (2012). Harnessing the mesenchymal stem cell secretome for the treatment of cardiovascular disease. Cell Stem Cell 10, 244–258. 10.1016/j.stem.2012.02.005 22385653 PMC3294273

[B77] Raser J. M. O'Shea E. K. (2005). Noise in gene expression: origins, consequences, and control. Science 309, 2010–2013. 10.1126/science.1105891 16179466 PMC1360161

[B78] Ratajczak M. Z. Zuba-Surma E. K. Wysoczynski M. Ratajczak J. Kucia M. (2008). Very small embryonic-like stem cells: characterization, developmental origin, and biological significance. Exp. Hematol. 36, 742–751. 10.1016/j.exphem.2008.03.010 18474305 PMC2430762

[B79] Ringden O. Uzunel M. Rasmusson I.. (2006). Mesenchymal stem cells for treatment of therapyresistant graft-versus-host disease. Transplantation 81, 1390–1397. 10.1097/01.tp.0000214462.63943.14 16732175

[B80] Russell K. C. Phinney D. G. Lacey M. R. Barrilleaux B. L. Meyertholen K. E. O'Connor K. C. (2010). *In vitro* high-capacity assay to quantify the clonal heterogeneity in trilineage potential of mesenchymal stem cells reveals a complex hierarchy of lineage commitment. Stem Cells 28, 788–798. 10.1002/stem.312 20127798

[B81] Russell K. C. Tucker H. A. Bunnell B. A. Andreeff M. Schober W. Gaynor A. S.. (2013). Cell-surface expression of neuron-glial antigen 2 (NG2) and melanoma cell adhesion molecule (CD146) in heterogeneous cultures of marrow-derived mesenchymal stem cells. Tissue Eng. Part A 19, 2253–2266. 10.1089/ten.tea.2012.0649 23611563 PMC3761443

[B82] Sacchetti B. Funari A. Michienzi S. Di Cesare S. Piersanti S. Saggio I.. (2007). Selfrenewing osteoprogenitors in bone marrow sinusoids can organize a hematopoietic microenvironment. Cell 131, 324–336. 10.1016/j.cell.2007.08.025 17956733

[B83] Sarugaser R. Hanoun L. Keating A. Stanford W. L. Davies J. E. (2009). Human mesenchymal stem cells self-renew and differentiate according to a deterministic hierarchy. PLoS ONE 4:e6498. 10.1371/journal.pone.0006498 19652709 PMC2714967

[B84] Schneider C. K. Salmikangas P. Jilma B. Flamion B. Todorova L. R. Paphitou A. Haunerova I.. (2010). Challenges with advanced therapy medicinal products and how to meet them. Nat. Rev. Drug Discov. 9, 195–201. 10.1038/nrd3052 20190786

[B85] Sengers B. G. Dawson J. I. Oreffo R. O. (2010). Characterisation of human bone marrow stromal cell heterogeneity for skeletal regeneration strategies using a two-stage colony assay and computational modelling. Bone 46, 496–503. 10.1016/j.bone.2009.10.002 19818885

[B86] Sensebé L. (2013). Beyond genetic stability of mesenchymal stromal cells. Cytotherapy 15, 1307–1308. 10.1016/j.jcyt.2013.09.001 24094485

[B87] Shabbir A. Zisa D. Suzuki G. Lee T. (2009). Heart failure therapy mediated by the trophic activities of bone marrow mesenchymal stem cells: a noninvasive therapeutic regimen. Am. J. Physiol. Heart Circ. Physiol. 296, H1888–H1897. 10.1152/ajpheart.00186.2009 19395555 PMC2716100

[B88] Sharma R. R. Pollock K. Hubel A. McKenna D. (2014). Mesenchymal stem or stromal cells: a review of clinical applications and manufacturing practices. Transfusion 54, 1418–1437. 10.1111/trf.12421 24898458 PMC6364749

[B89] Shoshani O. Ravid O. Massalha H. Aharonov A. Ovadya Y. Pevsner-Fischer M.. (2014). Cell isolation induces fate changes of bone marrow mesenchymal cells leading to loss or alternatively to acquisition of new differentiation potentials. Stem Cells 32, 2008–2020. 10.1002/stem.1719 24715711

[B90] Siegel G. Kluba T. Hermanutz-Klein U. Bieback K. Northoff H. Schäfer R. (2013). Phenotype, donor age and gender affect function of human bone marrow-derived mesenchymal stromal cells. BMC Med. 11:146. 10.1186/1741-7015-11-146 23758701 PMC3694028

[B91] Strioga M. Viswanathan S. Darinskas A. Slaby O. Michalek J. (2012). Same or not the same? Comparison of adipose tissue-derived versus bone marrow-derived mesenchymal stem and stromal cells. Stem Cells Dev. 21, 2724–5272. 10.1089/scd.2011.0722 22468918

[B92] Tarte K. Gaillard J. Lataillade J. J. Fouillard L. Becker M. Mossafa H.. (2010). Clinical-grade production of human mesenchymal stromal cells: occurrence of aneuploidy without transformation. Blood 115, 1549–1553. 10.1182/blood-2009-05-219907 20032501

[B93] Teles J. Pina C. Edén P. Ohlsson M. Enver T. Peterson C. (2013). Transcriptional regulation of lineage commitment—a stochastic model of cell fate decisions. PLoS Comput. Biol. 9:e1003197. 10.1371/journal.pcbi.1003197 23990771 PMC3749951

[B94] Thirumala S. Goebel W. S. Woods E. J. (2013). Manufacturing and banking of mesenchymal stem cells. Expert Opin. Biol. Ther. 13, 673–691. 10.1517/14712598.2013.763925 23339745

[B95] Tormin A. Li O. Brune J. C. Walsh S. Schütz B. Ehinger M.. (2011). CD146 expression on primary nonhematopoietic bone marrow stem cells is correlated with *in situ* localization. Blood 117, 5067–5077. 10.1182/blood-2010-08-304287 21415267 PMC3109533

[B96] Torres-Padilla M. E. Chambers I. (2014). Transcription factor heterogeneity in pluripotent stem cells: a stochastic advantage. Development 141, 2173–2181. 10.1242/dev.102624 24866112

[B97] Trombi L. Pacini S. Montali M. Fazzi R. Chiellini F. Ikehara S.. (2009). Selective culture of mesodermal progenitor cells. Stem Cells Dev. 18, 1227–1234. 10.1089/scd.2009.0054 19331526

[B98] Tyndall A. (2011). Successes and failures of stem cell transplantation in autoimmune diseases. Hematology Am. Soc. Hematol. Educ. Program. 2011, 280–284. 10.1182/asheducation-2011.1.280 22160046

[B99] Tyndall A. Walker U. A. Cope A. Dazzi F. De Bari C. Fibbe W.. (2007). Immunomodulatory properties of mesenchymal stem cells: a review based on an interdisciplinary meeting held at the Kennedy Institute of Rheumatology Division, London, UK, 31 October 2005. Arthritis Res. Ther. 9, 301. 10.1186/ar2103 17284303 PMC1860056

[B100] Wagner W. Ho A. D. (2007). Mesenchymal stem cell preparations—comparing apples and oranges. Stem Cell Rev. 3, 239–248. 10.1007/s12015-007-9001-1 18074246

[B101] Wang Y. Zhang Z. Chi Y. Zhang Q. Xu F. Yang Z.. (2013). Long-term cultured mesenchymal stem cells frequently develop genomic mutations but do not undergo malignant transformation. Cell Death Dis. 4, e950. 10.1038/cddis.2013.480 24309937 PMC3877551

[B102] Werts E. D. DeGowin R. L. Knapp S. K. Gibson D. P. (1980). Characterization of marrow stromal (fibroblastoid) cells and their association with erythropoiesis. Exp. Hematol. 8, 423–433. 7461050

[B103] W.H.O. (World Health Organization). (1997). A WHO guide to Good Manufacturing Practice (GMP). Published in 1997

[B104] Wu J. Tzanakakis E. S. (2012). Contribution of stochastic partitioning at human embryonic stem cell division to NANOG heterogeneity. PLoS ONE 7:e50715. 10.1371/journal.pone.0050715 23226362 PMC3511357

[B105] Yamada Y. Sakurada K. Takeda Y. Gojo S. Umezawa A. (2007). Single-cell-derived mesenchymal stem cells overexpressing Csx/Nkx2.5 and GATA4 undergo the stochastic cardiomyogenic fate and behave like transient amplifying cells. Exp. Cell Res. 313, 698–706. 10.1016/j.yexcr.2006.11.012 17208226

[B106] Yeatts A. B. Choquette D. T. Fisher J. P. (2013). Bioreactors to influence stem cell fate: augmentation of mesenchymal stem cell signaling pathways via dynamic culture systems. Biochim. Biophys. Acta 1830, 2470–2480. 10.1016/j.bbagen.2012.06.007 22705676 PMC3461086

[B107] Yeatts A. B. Fisher J. P. (2011). Bone tissue engineering bioreactors: dynamic culture and the influence of shear stress. Bone 48, 171–181. 10.1016/j.bone.2010.09.138 20932947

[B108] Ylöstalo J. Bazhanov N. Prockop D. J. (2008). Reversible commitment to differentiation by human multipotent stromal cells in single-cell-derived colonies. Exp. Hematol. 36, 1390–1402. 10.1016/j.exphem.2008.05.003 18619725 PMC2628773

[B109] Zhao R. C. (2013). Essentials of mesenchymal stem cell biology and its clinical translation. Springer Sci. Bus. Media 17–32. 10.1007/978-3-642-44910-9

[B110] Zuk P. A. Zhu M. Mizuno H. Huang J. Futrell J. W. Katz A. J.. (2001). Multilineage cells from human adipose tissue: implications for cell-based therapies. Tissue Eng. 7, 211–228. 10.1089/107632701300062859 11304456

